# Supplementary material for: Children with Hirschsprung’s Disease Report Dietary Effects on Gastrointestinal Complaints More Frequently than Controls
Source: Children (Basel). 2023 Sep 12;10(9):1543. doi: 10.3390/children10091543 (PMC10530174; doi:10.3390/children10091543)
Supplement: Supplementary file 1 [file children-10-01543-s001.zip › children-2574910-supplementary.pdf]

Supplementary Material

Table S1. Dietary effects on gastrointestinal symptoms and dietary adjustments adopted to improve gastrointestinal complaints reported by children aged 1-18 years with Hirschsprung's disease with different aganglionic extensions, Bowel Function Score, age and gender using the Diet and Bowel function questionnaire.

	HD rectosigmoid extension n=58	HD Long-segment aganglionosis n=13	p-value	HD BFS 1-17 n=35	HD BFS 18-20 n=15	p-value	HD 1-3 y n=15	HD 4-10 y n=40	HD 11-18 y n=16	p-value	HD girls n=15	HD boys n=56	p-value
**Diet affects GI symptoms**													
No	15 (26)	1 (8)		8 (23)	3 (20)		4 (27)	9 (23)	3 (19)		3 (20)	13 (23)	
Sometimes	23 (40)	6 (46)		14 (40)	7 (47)		5 (33)	18 (45)	6 (38)		5 (33)	24 (43)	
Often	10 (17)	3 (23)	0.227[1]	7 (20)	3 (20)	0.911[1]	3 (20)	7 (18)	3 (19)	0.741[3]	5 (33)	8 (14)	0.652[1]
Always	10 (17)	3 (23)		6 (17)	2 (13)		3 (20)	6 (15)	4 (25)		2 (13)	11 (20)	
Missing	0 (0)	0 (0)		0 (0)	0 (0)		0 (0)	0 (0)	0 (0)		0 (0)	0 (0)	
**Adjusts diet to improve GI symptoms**													
No adjustments	18 (31)	4 (31)		8 (23)	7 (47)		4 (27)	13 (33)	5 (31)		4 (27)	18 (32)	
Sometimes	19 (33)	4 (31)		13 (37)	3 (20)		5 (33)	13 (33)	5 (31)		5 (33)	18 (32)	
Often	8 (14)	3 (23)	0.938[1]	8 (23)	1 (7)	0.403[1]	2 (13)	7 (18)	2 (13)	0.848[3]	4 (27)	7 (13)	0.907[1]
Always	13 (22)	2 (15)		6 (17)	4 (27)		4 (27)	7 (18)	4 (25)		2 (13)	13 (23)	
Missing	0 (0)	0 (0)		0 (0)	0 (0)		0 (0)	0 (0)	0 (0)		0 (0)	0 (0)	
**Chooses food to improve GI complaints**													
Yes	19 (33)	8 (62)	0.053[2]	14 (40)	5 (33)	0.656[2]	6 (40)	17 (43)	4 (25)	0.468[2]	7 (47)	20 (36)	0.438[2]
No	39 (67)	5 (38)		21 (60)	10 (67)		9 (60)	23 (57)	12 (75)		8 (53)	36 (64)	
Missing	0 (0)	0 (0)		0 (0)	0 (0)		0 (0)	0 (0)	0 (0)		0 (0)	0 (0)	
**Avoids food to improve GI complaints**													
Yes	35 (60)	9 (69)	0.551[2]	25 (71)	6 (40)	0.036[2]	9 (60)	28 (70)	7 (44)	0.185[2]	7 (47)	37 (66)	0.169[2]
No	23 (40)	4 (31)		10 (29)	9 (60)		6 (40)	12 (30)	9 (56)		8 (53)	19 (34)	
Missing	0 (0)	0 (0)		0 (0)	0 (0)		0 (0)	0 (0)	0 (0)		0 (0)	0 (0)	

[1]Mann-Whitney U-Tests
[2]Chi-square Test
[3]Kruskal Wallis ANOVA
HD = Hirschsprung's disease
BFS = Rintala Bowel Function Score
GI = gastrointestinal

Table S2. Bowel function in children aged 4-18 years old with Hirschsprung's disease with different aganglionic extensions; rectosigmoid extension and long-segment aganglionosis, according to the Rintala Bowel Function Score (BFS). Numbers are (percentage) or as median (range).

	Score	HD Rectosigmoid extension n=45	HD Long-segment aganglionosis n=11	p-value[1]
**Ability to hold back defecation**				
Always	3	25 (56)	4 (44)	0.808
Problems <1/week	2	10 (22)	4 (44)	
Problems >1/week	1	4 (9)	0 (0)	
Never	0	6 (13)	1 (11)	
Missing		0 (0)	2 (18)	
**Feels the urge to defecate**				
Always	3	27 (60)	3 (33)	0.180
Often	2	7 (16)	3 (33)	
Sometimes	1	10 (22)	2 (22)	
Never	0	1 (2)	1 (11)	
Missing		0 (0)	2 (18)	
**Frequency of defecation**				
Every other day – twice a day	2	28 (62)	4 (44)	0.326
More often	1	17 (38)	5 (56)	
Less often	1	0 (0)	0 (0)	
Missing		0 (0)	2 (18)	
**Soiling**				
Never	3	13 (30)	1 (11)	0.200
Sometimes (<1/week)	2	14 (32)	2 (22)	
Often	1	12 (27)	5 (56)	
Always	0	5 (11)	1 (11)	
Missing		1 (2)	2 (18)	
**Accidents**				
Never	3	31 (69)	4 (44)	0.244
Sometimes (<1/week)	2	8 (18)	4 (44)	
Often (>1 week)	1	2 (4)	0 (0)	
Daily	0	4 (9)	1 (11)	
Missing		0 (0)	2 (18)	
**Constipation**				
No constipation	3	29 (66)	8 (80)	0.383
Manageable with diet	2	2 (5)	0 (0)	
Manageable with medicine	1	10 (23)	2 (20)	
Not manageable with either diet or medicine	0	3 (7)	0 (0)	
Missing		1 (2)	1 (9)	
**Social problems**				
No social problems	3	25 (57)	4 (36)	0.106

Sometimes	2	12 (27)	2 (18)	
Problems causing restrictions in social life	1	6 (14)	5 (46)	
Severe social and/or psychological problems	0	1 (2)	0 (0)	
Missing		1 (2)	0 (0)	
**Total Bowel Function Score**		n=42	n=8	
BFS	1-20	16 (4-20)	15 (9-20)	0.631

[1]Mann-Whitney U-Tests, two-tailed.
HD = Hirschsprung's disease.

Table S3. Self-reported gastrointestinal symptoms in children aged 1-18 years old with Hirschsprung's disease with different aganglionic extensions; rectosigmoid extension and long-segment aganglionosis, using the Diet and Bowel Function questionnaire. Numbers are (percentage) or as median (range).

	HD Rectosigmoid extension n=58	HD Long-segment aganglionosis n=13	p-value
**Constipation**			
No constipation	36 (63)	10 (83)	0.183[1]
Constipation without treatment	1 (2)	0 (0)	
Manageable with diet	2 (4)	0 (0)	
Manageable with medicine	14 (25)	2 (17)	
Not manageable with either diet or medicine	4 (7)	0 (0)	
Missing	1 (2)	1 (8)	
**Abdominal pain**			
Never	19 (33)	3 (23)	0.802[1]
Sometimes (at most once a week)	27 (47)	8 (62)	
Often	12 (20)	2 (15)	
Always	0 (0)	0 (0)	
Missing	0 (0)	0 (0)	
**Bothersome gases**			
Never	6 (10)	0 (0)	0.923[1]
Sometimes (at most once a week)	21 (36)	7 (54)	
Often	26 (45)	5 (39)	
Always	5 (9)	1 (8)	
Missing	0 (0)	0 (0)	
**GI symptoms**			
No/Seldom GI symptoms	16 (28)	4 (31)	0.818[2]
GI symptoms (Often/Always)	42 (72)	9 (69)	

[1]Mann-Whitney U-Tests, two-tailed.
[2]Chi-square Tests
HD = Hirschsprung's disease.

Table S4. Dietary effects on daily life in children aged 1-18 years old with and without Hirschsprung's disease using the Diet and Bowel Function questionnaire.
Values are n (%) of children.

	All children n=336			Children with GI symptoms n=128		
	HD n=71	Control n=265	p-value	HD n=51	Control n=77	p-value
**Diet causes social problems**						
No	28 (58)	99 (82)	0.002[1]	19 (54)	38 (73)	0.004[1]
Sometimes	16 (33)	17 (14)		12 (34)	11 (21)	
Often	3 (6)	2 (2)		3 (9)	1 (2)	
Always	1 (2)	3 (3)		1 (3)	2 (4)	
Missing	23 (32)	144 (54)		16 (31)	25 (33)	
**Children: Concerned about diet's impact on GI complaints**						
No effects	42 (62)	204 (77)	0.003[1]	27 (56)	48 (62)	0.130[1]
Sometimes	16 (24)	51 (19)		14 (29)	21 (27)	
Often	6 (9)	6 (2)		5 (10)	6 (8)	
Always	4 (6)	3 (1)		2 (4)	2 (3)	
Missing	3 (4)	1 (0)		3 (6)	0 (0)	
**Parents: Concerned about diet's impact on their child's GI complaints**						
No adjustments	8 (11)	95 (37)	<0.001[1]	3 (6)	20 (27)	0.003[1]
Sometimes	22 (31)	97 (38)		16 (32)	28 (37)	
Often	17 (24)	39 (15)		13 (26)	15 (20)	
Always	23 (33)	25 (10)		18 (36)	12 (16)	
Missing	1 (1)	9 (3)		1 (2)	2 (3)	
**Children: Emotional effects of diet**						
No thoughts	48 (68)	216 (83)	0.004[1]	30 (63)	61 (81)	0.273[1]
Sometimes	17 (25)	32 (12)		13 (27)	8 (11)	
Often	4 (6)	9 (4)		4 (8)	4 (5)	
Always	1 (2)	2 (1)		1 (2)	2 (3)	
Missing	3 (4)	6 (2)		3 (6)	2 (3)	
**Parents: Emotional effects of diet**						
No thoughts	34 (49)	187 (73)	<0.001[1]	21 (42)	49 (66)	<0.001[1]
Sometimes	21 (30)	46 (18)		19 (38)	21 (28)	
Often	8 (11)	17 (7)		5 (10)	4 (5)	
Always	7 (10)	5 (2)		5 (10)	0 (0)	
Missing	1 (1)	10 (4)		1 (2)	3 (4)	
**Interest in improving diet literacy**						
Yes	43 (61)	55 (21)	<0.001[2]	32 (63)	19 (25)	<0.001[2]
No	28 (39)	208 (79)		19 (37)	58 (75)	
Missing	0 (0)	2 (1)		0 (0)	0 (0)	

[1]Mann-Whitney U-Test
[2]Chi-square test
HD = Hirschsprung's disease; GI = gastrointestinal.

Background data	Gastrointestinal symptoms and Bowel Function Score	Dietary habits and gastrointestinal symptoms	Specific food items and gastrointestinal symptoms
**1. Age**<br>(Years)<br>**2. Gender**<br>(Girl/Boy)<br>**3. Height**<br>(cm)<br>**4. Weight**<br>(kg)<br>**5a. Do you have any disease, allergy or any gastrointestinal congenital malformation?**<br>(Yes/No)<br>**5b. If yes, What allergy/disease?**<br>(Open answer)<br>**5c. What is the gastrointestinal congenital malformation?**<br>(Open answer)<br>**6a. Do you take any treatment for bowel symptoms?**<br>(Yes/No)<br>**6b. If yes, What type of treatment?**<br>(Adjusted diet/Medicine (e.g. for diarrhea or constipation, antibiotics, analgesic), What type of medicine?/Enemas/Other reason)<br>**6c. If yes, for what reason?**<br>(Constipation/Diarrhea/Stomach pain/Other reason)<br>**7. Have you ever had impaired growth?** (Weight Yes/No, Height Yes/No)<br>**8. Have you ever had the need for nutritional supplements**<br>**(e.g. oral nutritional supplements)?**<br>(No, never, Not currently, but I have in the past, Yes, occasionally need, Yes, need regularly)<br>**9a. Do you have any special diet? (e.g. vegetarian, vegan)**<br>(Yes/no)<br>**9b. If yes, What type of special diet?**<br>(Open answer)	**10a. How often do you/your child poop?** (Every other day to twice a day/More often than twice a day/Less often than every other day)<br>**10b. How many times a week do you/your child poop?**<br>(Open answer)<br>**11. Do you/does your child have hard poop and troubles to push it out?** (No/ Yes, but it passes without doing anything/ Yes, then I need to eat prunes, pears, kiwis or other food to make the poop more soft/ Yes, I need to eat medicine that makes the poop softer/ Yes, I need to use medicine in my butt to be able to push the poop out)<br>**12. Do you/does your child ever experience difficulties in school or in their spare time, for example doing what you/your child wants, or seeing friends, due to problems with stools coming in underwear or the risk of it happening?** (No social problems/ Sometimes/ Often/ Daily)<br>**13. Do you/does your child feel the urge to poop and verbalizes when you/he or she will poop?**<br>(Always/Most of the time/Not often/seldom/Never)<br>**14. Are you/your child able to hold back defecation?**<br>(Yes, always or almost always/ Problems less than 1 time per week/ Weekly/ No, my child has no or little voluntary control)<br>**15. Do you/does your child ever soil (staining in underwear)?** (Never or very seldom/Less than 1 time per week no change of underwear required/Often, change of underwear required many times per week/Daily, requires protective aids)<br>**16. Do you/does your child ever have accidents where a lot of poop comes out in the underwear?**<br>(Never/Less than 1 time per week/Weekly, often require protective aids/Daily, protective aids required day and night)<br>**17. Do you experience abdominal pain?** (Never/ Sometimes [at most once a week]/ Frequently/Always)<br>**18. Do you suffer from bothersome gases?** (Never/ Sometimes [at most once a week]/Frequently/Always)<br>**19. Do you experience difficulties in passing stools despite soft poop?** (No, never/Yes, without treatment/Yes, manageable with diet/Yes, manageable with medicine/Yes, not manageable with either diet and/or medicine)	**20. Would you agree that your diet affects your stomach?**<br>**(e.g. constipation, diarrhea or bloatedness)**<br>(No, never/Yes, sometimes/Yes, often/Yes, always/Not currently but I have in the past/Please explain how)<br>**21. Would you agree that how you eat affects your stomach?**<br>(No, never/Yes, sometimes/Yes, often/Yes, always/Not currently but I have in the past/Please explain how)<br>**22a. Do you adjust your diet for your stomach's sake?**<br>(No, never/Yes, sometimes/Yes, often/Yes, always/Not currently but I have in the past/Please explain how)<br>**22b. If yes: Why?**<br>(Laxative effect/Constipating effect/Less gases/Other. If so, what?)<br>**23. Do you choose specific types of food to help your stomach?**<br>(Yes/No)<br>**24. Do you avoid specific types of food to help your stomach?**<br>(Yes/No)<br>**25. Is there anyone else in your family who adjusts their diet to help their stomach?**<br>(Yes/No)<br>**26. Does your diet limit you (in school, when you are with friends or in general)?**<br>(I've never thought about it, so I don't find it relevant/No, never/Yes, sometimes/Yes, often/Yes, always/Not currently but I have in the past/Please explain how)<br>**27. Do you think about how your diet affects your stomach?**<br>(No, never/Yes, sometimes/Yes, often/Yes, always/Not currently but I have in the past/Please explain how)<br>**28. To parents: Do you think about your child's diet and how it affects his/her stomach?**<br>(No, never/Yes, sometimes/Yes, often/Yes, always/Not currently but I have in the past/Please explain how)<br>**29. Does your diet affect you emotionally?**<br>(No, never/Yes, sometimes/Yes, often/Yes, always/Not currently but I have in the past/Please explain how)<br>**30. To parents: Does your child's diet affect you emotionally?**<br>(No, never/Yes, sometimes/Yes, often/Yes, always/Not currently but I have in the past/Please explain how)<br>**31a. Would you be interested in finding out more information about how your diet affects your stomach?**<br>(Yes/No)<br>**31b. If yes: Where or who would you turn to to find out more information?**<br>(Open answer)	**32a. Does the food item affect your stomach?**<br>(For every food item: Yes/No/I don't know)<br>**32b. If yes: In which way?**<br>(For every food item: Laxative effect/Constipating effect/Gives gases/Gives pain/Other. If so, what?)<br><br>**Food items listed:**<br>**Fruits**: pineapple, orange, apricot, banana, clementine, strawberry, kiwi, cherry, lingonberry, melon, nectarine, peach, plum, pear, dried fruit, grapes, apple<br>**Vegetables**: avocado, cauliflower, broccoli, beans, cabbage, lentils, onion, corn, carrot, pepper, parsley, potato, rhubarb, celery, fruit peel, asparagus, mushroom, tomato, peas<br>**Dairy**: cream, ice cream, lactose-free milk, milk, cheese, butter, yoghurt<br>**Bread, flour, rice**: bread with grains/seeds, Swedish cracker, cornmeal, pasta, rice, flour, white bread<br>**Meat, fish, egg**: fish, pork, beef, sausage, chicken, salami, shellfish, egg<br>**Sweets and snacks**: pastry, chips, chocolate, candy, popcorn, rice cakes, pretzel sticks<br>**Beverages**: soda (with sugar), soda (free from sugar), water, carbonated drink, wine/beer, formula<br>**Cooking effects**: deep fried food, spicy food, fried food, soup<br>**Spices and seeds**: curry, cayenne, chili/tabasco, chamomilla, sesame, sunflower seeds, poppy seeds<br>**Nuts etc**: cashew, hazelnut, peanut, chestnut, almond, brazil nut, walnut, soy

Figure S1. The Diet and Bowel function questionnaire. Questions and answering options.

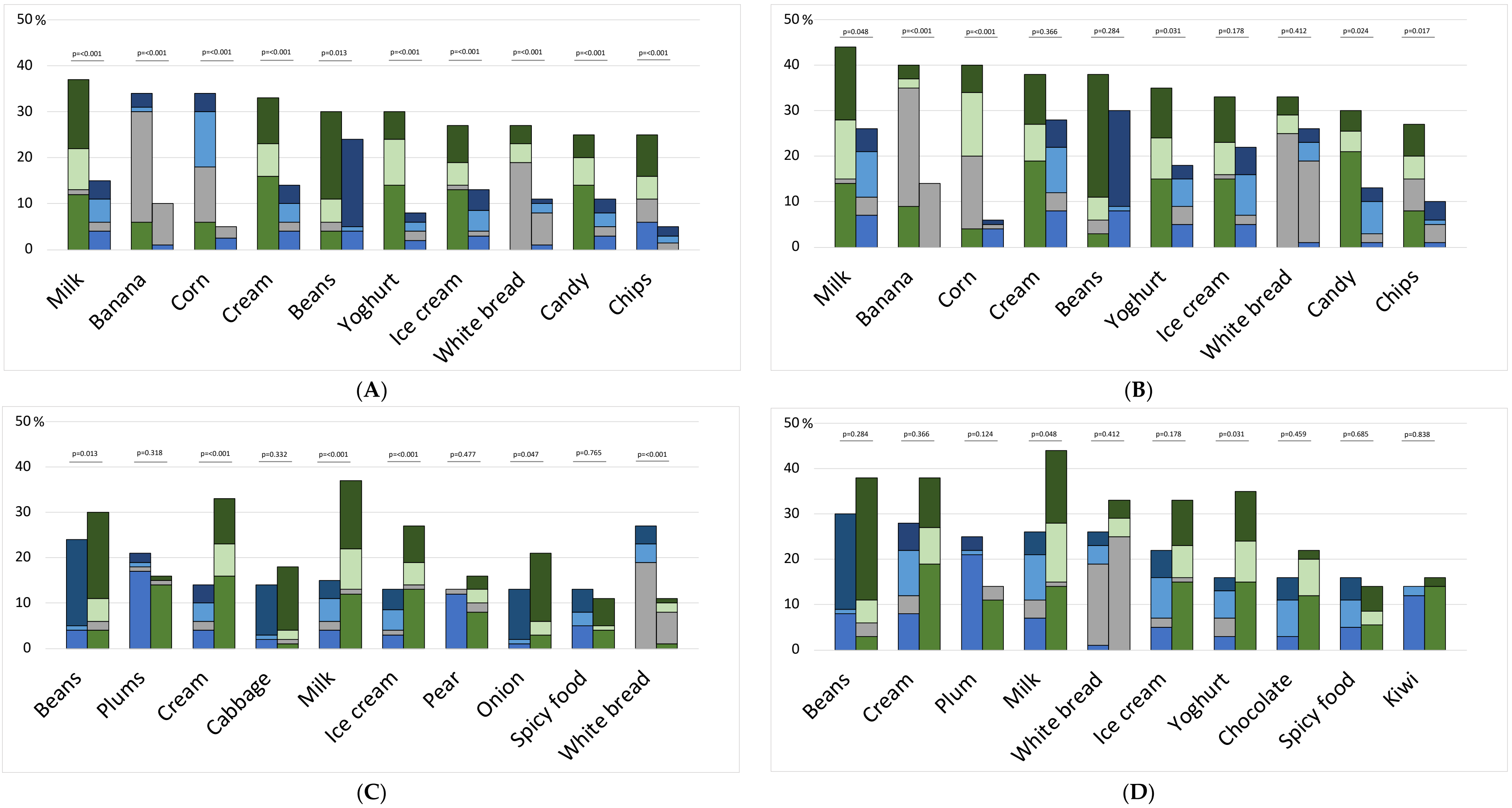

Figure S2. (**A–D**). Frequencies of the 10 most reported food items to induce GI effects and their specific effects in (**A**) children with Hirschsprung's disease (n=71); (**B)** children with Hirschsprung disease with gastrointestinal complaints (n=51); (**C**) healthy children (n=265) and (**D**) healthy children with gastrointestinal complaints (n=77) using the Diet and Bowel Function questionnaire.
